# Dietary Conjugated Linoleic Acid Supplementation Leads to Downregulation of PPAR Transcription in Broiler Chickens and Reduction of Adipocyte Cellularity

**DOI:** 10.1155/2014/137652

**Published:** 2014-09-18

**Authors:** Suriya Kumari Ramiah, Goh Yong Meng, Tan Sheau Wei, Yeap Swee Keong, Mahdi Ebrahimi

**Affiliations:** ^1^Institute of Tropical Agriculture, Universiti Putra Malaysia, Persiaran UPM Serdang, 43400 Serdang, Selangor, Malaysia; ^2^Faculty of Veterinary Medicine, Universiti Putra Malaysia, Persiaran UPM Serdang, 43400 Serdang, Selangor, Malaysia; ^3^Institute of Bioscience, Universiti Putra Malaysia, Persiaran UPM Serdang, 43400 Serdang, Selangor, Malaysia

## Abstract

Conjugated linoleic acids (CLA) act as an important ligand for nuclear receptors in adipogenesis and fat deposition in mammals and avian species. This study aimed to determine whether similar effects are plausible on avian abdominal fat adipocyte size, as well as abdominal adipogenic transcriptional level. CLA was supplemented at different levels, namely, (i) basal diet without CLA (5% palm oil) (CON), (ii) basal diet with 2.5% CLA and 2.5% palm oil (LCLA), and (iii) basal diet with 5% CLA (HCLA).The content of* cis*-9,* trans*-11 CLA was between 1.69- and 2.3-fold greater (*P* < 0.05) than that of* trans*-10,* cis*-12 CLA in the abdominal fat of the LCLA and HCLA group. The adipogenic capacity of the abdominal fat depot in LCLA and HCLA fed chicken is associated with a decreased proportion of adipose cells and monounsaturated fatty acids (MUFA). The transcriptional level of adipocyte protein (aP2) and peroxisome proliferator-activated receptor gamma (PPAR*γ*) was downregulated by 1.08- to 2.5-fold in CLA supplemented diets, respectively. It was speculated that feeding CLA to broiler chickens reduced adipocyte size and downregulated PPAR*γ* and aP2 that control adipocyte cellularity. Elevation of CLA isomers into their adipose tissue provides a potential CLA-rich source for human consumption.

## 1. Introduction

Modern bird strains tend to accumulate excess fat [1]. This tendency has proven to be one of the main problems for poultry producers as it has a negative effect on the broiler industry today because excessive fat deposits result in lower meat yields [2]. There are evidences that the growth pattern of fat depots in chickens can be affected by dietary factors [3–5]. CLA is a mixture of mainly* cis*-9,* trans*-11 and* trans*-10,* cis*-12 isomers. Numerous biological effects of CLA have been described [6–9].* In vivo* and* in vitro* studies showed that the inclusion of CLA in the diet modulates cell growth, nutrient utilization, nutrient storage, and lipid metabolism mainly in rodents and pigs [10, 11]. Many studies also confirmed that CLA are able to modulate fat deposition patterns in chickens [12–14].

The transcription level of adipogenic genes in adipose tissues is regulated by a number of transcription factors [15], whose differential transcription is known to play a key role in lipid metabolism of poultry adipocytes [16]. PPAR receptors belong to the nuclear receptor superfamily. Three subtypes of PPAR have been identified, which are PPAR*α*, PPAR*δ*, and PPAR*γ* [17]. Houseknecht et al. [18] first reported the antidiabetic effects of dietary CLA and the link to PPAR*γ* in rats. In mammals, PPAR*γ* is highly expressed in adipose tissue [19]. Study by Larkina et al. [20] showed that PPAR*γ* transcription was induced highly in liver of fatty chicken, but not in the adipose. This could be due to a divergence of PPAR signal transduction mechanisms between avian and mammals.

PPAR*γ* stimulate anabolic processes such as triglyceride synthesis, glucose uptake, and fatty acid uptake by directly or indirectly regulating target genes such as aP2, lipoprotein lipase (LPL), GLUT4, and the fatty acid translocase (CD-36/FAT) [21]. Kang et al. [22] reported that* trans*-10,* cis*-12 CLA reduced the transcription levels of the adipocyte transcription factor PPAR*γ*, adipocyte gene fatty acid synthase (FASN), and aP2 compared to vehicle-treated control group in* 3T3-L1* adipocytes cells.

Excessive fat deposition is highly correlated with increased adipocyte size. Both* in vitro* and* in vivo* studies in monogastric species demonstrated that CLA decreases adipocyte cellularity by decreasing adipocyte proliferation [23, 24] or adipocyte size [24–26]. In rats, CLA have been reported to reduce the adipocyte size and diameter in rats [27]. In fact, CLA-induced decrease in body fat mass in rats was due to a decrease in adipocyte size, rather than adipocyte number [25]. Similarly, Barnes et al. [28] indicated that the increase in intramuscular fat in the CLA-fed pigs appears to be associated with a greater increase in intramuscular adipocyte size than the number. In contrast, in CLA-fed cattle, the intramuscular adipose tissue was reported to increase via both hypertrophy and hyperplasia [29]. Therefore there could be species difference in adipocyte responses to presence of CLA in their cellular environment.

The adipose tissue depots in poultry may possess varied transcription and regulation of adipogenic state-specific genes, which result in differences in adipose accretion as growth and development progress. Studies have shown that smaller adipocytes have decreased FASN and LPL enzymatic activities that lead to less* de novo* synthesis of fatty acids and reduced uptake of lipoproteins for storage in rat and human [30]. FASN mRNA in human adipose tissue was shown to have positive correlation with insulin sensitivity and increased after treatment with pioglitazone (PPAR*γ* agonist) [31].

The current work reports on the body fat-decreasing effects of CLA in broiler chicken associated with adipogenic genes. The quantitative changes induced by CLA on adipocyte size and distribution will also be investigated. Therefore, we assessed the mean adipocyte areas (*μ*m^2^) of abdominal fat to characterize fat depot in terms of adipocyte size and to investigate whether and to what extent a CLA supplement changes the abdominal adipocyte size and related transcriptional markers in broiler chicken.

## 2. Materials and Methods

### 2.1. Experimental Birds and Diets

A total of 180-day-old male broiler chicks (Cobb 500) were obtained from a local hatchery. Upon arrival, the chicks were individually wing-tagged, weighed, and randomly assigned into three treatment groups. The birds received a starter feed from day 1 till day 21 and finisher feed between days 22 and 42. Each treatment group had six replicates of 10 birds and was raised in 18 battery cages with wire floors. The cages were in a conventional open-sided house with cyclic temperatures (minimum, 24°C; maximum, 34°C). The relative humidity was between 80 and 90%. Feed and water were provided* ad libitum* and lighting was continuous. The chicks were vaccinated against Newcastle disease on day 7. Commencing from day 1, six cages of birds were assigned to one of the 3 dietary groups: (i) basal diet (5% palm oil) without CLA, (ii) 2.5% CLA and 2.5% palm oil (LCLA: low CLA), and (iii) 5% CLA (HCLA: high CLA). The CLA used in this study was a commercial feed grade (Lutrell BSAF, SE, Ludwigshafen, Germany). The diets were in mash form. The composition of experimental diets was formulated to meet or exceed NRC [32] recommendations. Tables 1 and 2 show the chemical composition and fatty acid profile of the experimental diets, respectively. The three experimental diets, which were isocaloric, are shown in Table 1. The average metabolizable energy content ranged from 3080 to 3150 Mcal/kg of the dry matter (DM) content, whilst the protein content was 22% (of DM) for starter and 20.5% (of DM) for the finisher diet. The crude fat was 5% in all treatment groups.

### 2.2. Animal Welfare

This experimental protocol was undertaken following the guidelines of the Research Policy of the Universiti Putra Malaysia on animal ethics.

### 2.3. Total Lipid Extraction

Total lipid extraction was performed on the abdominal adipose tissues harvested from the experimental animals. The adipose tissues from abdominal cavity were obtained from 10 birds from each treatment group on day 42 after slaughter. The tissues were snap-frozen and stored at −20°C until further analyses. Total fatty acids from abdominal adipose tissue and feed were extracted using a chloroform-methanol (2 : 1 v/v) solvent system according to Folch et al. [33] and modified by Rajion et al. [34] as described by Ebrahimi et al. [35]. Briefly, fatty acid methyl esters (FAME) were prepared using 0.66 N potassium hydroxide (KOH) in methanol and 14% methanolic boron trifluoride (BF_3_) (Sigma Chemical Co., St. Louis, Missouri, USA).

### 2.4. Fatty Acid Analysis

Fatty acid methyl esters were separated and quantified by gas-liquid chromatography (Model 7890A, Agilent Technologies, USA) using a 100 m × 0.32 mm i.d. capillary column (SP-2560, Supelco, Inc., Bellefonte, PA, USA). The hydrogen was used as the carrier gas at 40 mL/min. The injector temperature was programmed at 250°C and the detector temperature was 300°C. The column temperature program initiated to run at 120°C, for 5 min, increased to 170°C at 2°C/min and held at 15 min and increased to 200°C by 5°C/min and then held at 200°C for 5 min and then warmed to 235°C for 2°C/min and held for 10 min. The identification of the peaks was made by comparing equivalent chain lengths with a reference standard (mix C4–C24 methyl esters; Sigma Aldrich, Inc., St. Louis, MO, USA) and CLA standard mix (*cis*-9,* trans*-11 CLA and* trans*-10,* cis*-12 CLA, Sigma-Aldrich, Inc, St. Louis, MO, USA). Peak areas were determined automatically using the Agilent gas chromatography Chemstation software (Agilent Technologies, USA) as described by Ebrahimi et al. [36]. The fatty acid concentrations are expressed as percentage of the sum of total identified peaks measured in each sample.

### 2.5. RNA Extraction

Samples from abdominal fat were collected, snap-frozen in liquid nitrogen, and stored at −80°C for RNA extraction. Total RNA extractions of each tissue sample were extracted using Qiagen, RNeasy Lipid Tissue Mini Kit according to the manufacturer's instruction. Total RNA concentration was then quantified by measuring the optical density. The ratios of absorption at 260/280 nm of all preparations were between 1.8 and 2.0.

### 2.6. Real-Time Polymerase Chain Reaction (PCR)

Five *μ*g of RNA from each sample was reverse-transcribed using the QuantiTect Rev Transcription Kit (Qiagen, Hilden, Germany) according to the manufacturer's protocol. Quantitative real-time PCR was performed to measure transcriptional level of PPAR*α*, PPAR*γ*, and aP2. *β*-actin and GAPDH were used as the reference gene for normalization. Primer sequences and optimal PCR annealing temperatures are listed in Table 3. These primers were purchased from 1st BASE Oligonucleotide Synthesis (1st Base, Singapore). The amplification procedure was performed in a 20 *μ*L reaction volume with final concentration of 1X QuantiFast SYBR green PCR kit (Qiagen, Hilden, Germany), 1 *μ*g cDNA, 400 nM/500 nM of each of the forward and reverse primers, and 8.5 *μ*L RNase free water. The following thermal-cycling conditions were used: PCR initial activation (10 min at 95°C) and 40 cycles of denaturation (15 sec at 95°C), annealing (20 sec at different temperature according to targeted genes listed in Table 3), and extension (20 sec at 72°C). The efficiency of each target and reference genes was validated by titrating respective cDNA template at six serial dilutions in the PCR experiment. Primer pairs were revalidated for those efficiency values below 90% and above 110%. For all target genes investigated in this study, optimum efficiencies between 94% and 110% for each of the respective primer pairs were obtained. Real-time PCR was performed using the Bio-Rad CFX96 Touch Real-Time PCR Systems (Bio-Rad, USA) on optical grade plates using QuantiFast SYBR green PCR kit (Qiagen, Hilden, Germany). Each sample was run in triplicate and averaged triplicates were used to assign Cq (quantification cycle) values. Template control was included in each run. A higher initial concentration correlates to a lower Cq value and thus has high transcription level. The transcription levels were calculated as described by Vandesompele et al. [37]. The real-time PCR data was normalized by geometric averaging of two reference genes, namely, GAPDH and *β*-actin, in this study. The transcription levels of PPAR*α*, PPAR*γ*, and aP2 in treated groups were compared to control groups. The transcription levels for the controls are always expressed as 1-fold.

### 2.7. Isolation and Culture of Adipocyte Cells

One gram of abdominal fat was taken from the broiler immediately after slaughter. Isolation of fat cell was done according to the method by Rodbell [38] with some modification by Tekeleselassie et al. [30]. The adipose was minced into small fragments using scissors. The minced fat tissue was placed in a plastic tube containing phosphate buffer saline (PBS) supplemented with 5 mL of 2 mg/mL of type II collagenase from* Clostridium hemolyticum* (Sigma, Sigma-Aldrich, St. Louis, MO, USA). The adipose tissue was incubated in a water bath at 37°C for 50 minutes with occasional shaking. At the end of incubation, undigested fibrous tissues were removed using forceps. In order to inhibit the collagenase activity, PBS was added and the suspension was centrifuged at 200 ×g for five minutes. The infranatant was removed by gentle aspiration using a plastic Pasteur pipette. The latter procedure was repeated twice. Finally, PBS was added to bring the suspension to a total volume of 5 mL for determination of the number and diameter of adipocyte cells.

### 2.8. Determination of Abdominal Fat Cellularity

Aliquots of suspended adipocytes were placed in a haemacytometer and observed under an Olympus microscope BX51 (Olympus, Tokyo, Japan) and photographs were taken using an image analysis software (cc-12 soft imaging system). Adipocytes were then enumerated and their diameters measured using the photographic image. The mean adipocyte diameter was calculated as an average of the diameters of 200 cells.

### 2.9. Statistical Analysis

Fatty acid data and abdominal fat and transcription data were analyzed using the multivariate analysis of variance (ANOVA) procedure of the SAS software package, version 9.1 (SAS Institute Inc., Cary, NC). Significantly different mean values were further elucidated using Duncan's test. The results were expressed as mean and pooled standard error of mean (SEM). Differences were considered to be statistically significant when *P* < 0.05.

## 3. Results

### 3.1. Fatty Acid Composition of the Abdominal Fat

Generally the fatty acids profile of the abdominal fat mirrors that of the dietary fatty acid profile with several notable differences as shown in Table 4. The content of* cis*-9,* trans*-11 CLA ranged from 1.69- to 2.3-fold greater (*P* < 0.05) than that of* trans*-10,* cis*-12 CLA in abdominal fat of LCLA and HCLA groups. The SFA was significantly higher in the HCLA group mainly due to the increase in the concentration of myristic (C14:0), palmitic (C16:0), and stearic (C18:0) acids. The unsaturated fatty acid in abdominal fat was found to be higher in the CON and LCLA groups compared to the HCLA animals. The MUFA in abdominal fat was significantly (*P* < 0.05) lower in the HCLA treatment group compared to CON and LCLA. The changes of MUFA in HCLA can be traced to the reduction in the concentration of palmitoleic (C16:1) and oleic acid (C18:1 n-9). However, total PUFA were not significantly (*P* > 0.05) different among the treatment diets. The ratios of n-6 : n-3 fatty acid, PUFA : SFA in the abdominal fat of HCLA treatment group were significantly lower compared to the CON and LCLA treatment groups. CLA isomers were not found in the adipose tissues of broilers fed with control diet. It was also noted that the CLA content of abdominal fat in chickens increased with the increasing doses of CLA sources from 0.00 (CON) to 1.30 (LCLA) and 4.07 (HCLA).

### 3.2. Abdominal Fat PPAR and aP2 Transcriptional Levels

**Figure 1:**
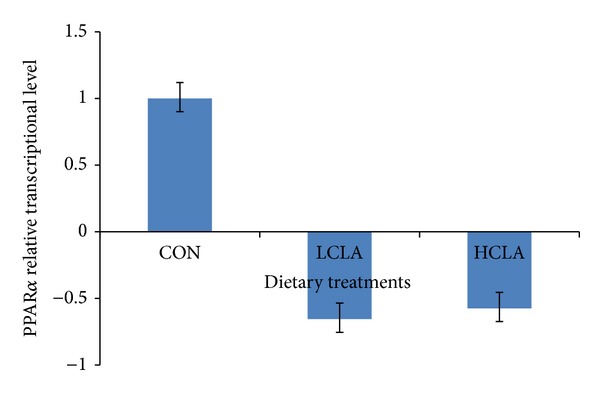
Comparison of PPAR*α* relative transcriptional level in the abdominal fat of chickens fed diets with CON, LCLA, and HCLA. Values were normalized with reference genes, *β*-actin, and GAPDH. Then, treated samples were expressed relative to transcriptional level of CON group. Values are mean ±1 standard error (*n* = 10). CON: without CLA; LCLA: low CLA; HCLA: high CLA. LCLA and HCLA found not to be of significant difference compared to CON group (*P* > 0.05).

**Figure 2:**
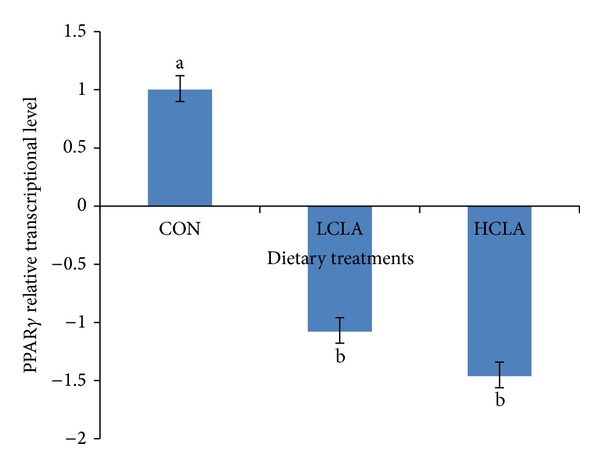
Comparison of PPAR*γ* relative transcriptional level in the abdominal fat of chickens fed diets with CON, LCLA, and HCLA. Values were normalized with reference genes, *β*-actin, and GAPDH. Then, treated samples were expressed relative to transcriptional level of CON group. Values are mean ±1 standard error (*n* = 10). CON: without CLA; LCLA: low CLA; HCLA: high CLA. Letter in superscript indicated a significant difference compared with the CON group (*P* < 0.05).

**Figure 3:**
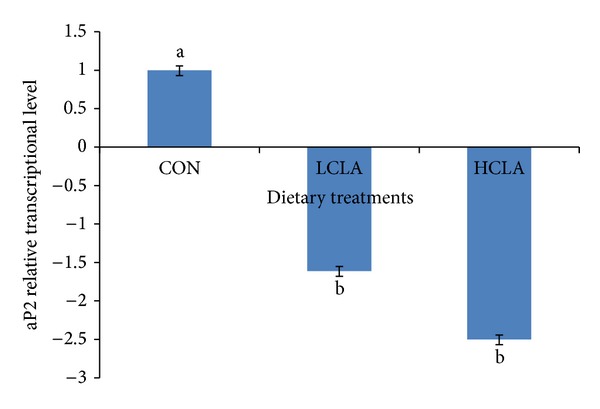
Comparison of aP2 relative transcriptional level in the abdominal fat of chickens fed diets with CON, LCLA, and HCLA. Values were normalized with reference genes, *β*-actin, and GAPDH. Then, treated samples were expressed relative to transcriptional level of CON group. Values are mean ±1 standard error (*n* = 10). CON: without CLA; LCLA: low CLA; HCLA: high CLA. Letter in superscript indicated a significant difference compared with the CON group (*P* < 0.05).

The changes to the PPAR*α*, PPAR*γ*, and aP2 genes in the adipose tissue of HCLA and LCLA dietary groups, compared to the CON group, are shown in Figures 1, 2, and 3, respectively. No significant differences (*P* > 0.05) in the level of PPAR*α* transcription in all treatment groups were observed (Figure 1). However, the PPAR*γ* and aP2 transcription were significantly lower (*P* < 0.05) in the LCLA and HCLA treated animals, vis-à-vis the control animals (CON) as shown in Figures 2 and 3, respectively. This observation indicates that PPAR*γ* and aP2 were downregulated between 1.08- and 2.5-fold by CLA dietary supplementation.

### 3.3. Abdominal Fat Cellularity

**Figure 4:**
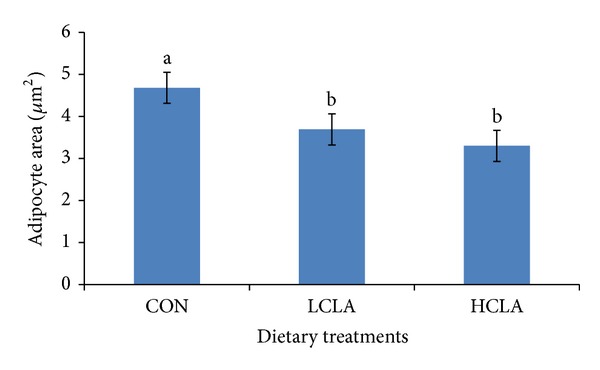
Effect of dietary conjugated linoleic acid (CLA) on adipocyte area distribution in the abdominal fat in chickens fed with CON (control), LCLA (low conjugated linoleic acid), and HCLA (high conjugated linoleic acid) for 6 weeks. Data represents the mean of cell area. Data are presented as mean ± SEM. Different superscripts within a cell size range denote significant differences (*P* < 0.05).

**Figure 5:**
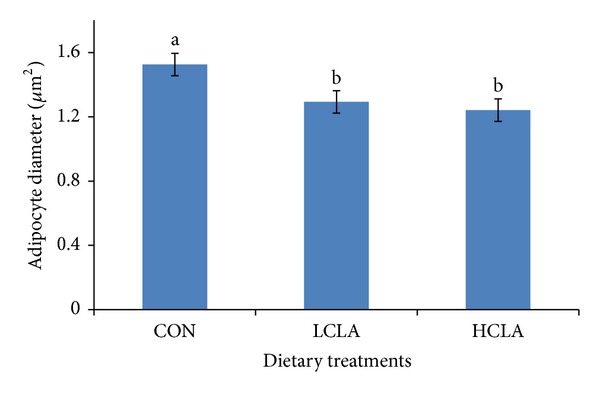
Effects of dietary conjugated linoleic acid (CLA) on adipocyte diameter in chickens fed with CON (control), LCLA (low conjugated linoleic acid), and HCLA (high conjugated linoleic acid) for 6 weeks. Data represent the mean of cell diameter. Data are presented as mean ± SEM. Different superscripts among bars denote significant differences (*P* < 0.05).

**Figure 6:**
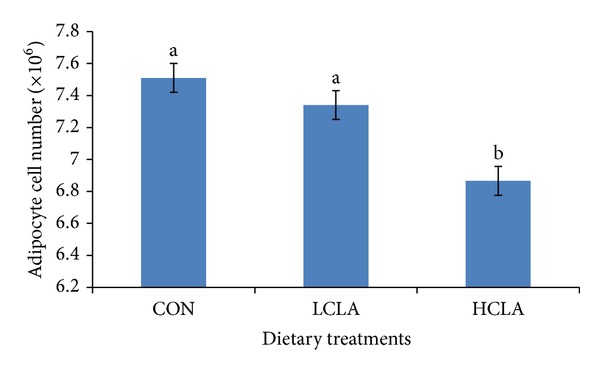
Effects of dietary conjugated linoleic acid (CLA) on adipocyte cell number in chickens fed with CON (control), LCLA (low conjugated linoleic acid), and HCLA (high conjugated linoleic acid) for 6 weeks. Data represents the mean of cell number. Data are presented as mean ± SEM. Different superscripts between bars denote significant differences (*P* < 0.05).

The area size, diameter, and number of abdominal adipocytes are shown in Figures 4, 5, and 6, respectively. The data clearly indicated that the type of dietary fat had a pronounced effect on fat cell cellularity. The mean adipocyte number per gram of abdominal fat in HCLA supplemented groups was significantly lower compared to the CON and LCLA groups. The mean diameter and area of abdominal adipocytes from the CLA-supplemented diet were significantly smaller than the CON group.

## 4. Discussion

Although a considerable amount is known about the effects of CLA on PPAR*γ* transcription and on its downstream target genes in rodents and humans, there is a scarcity of data examining PPAR*γ* transcription and function in the chicken's adipose. PPAR*γ* gene is shorter than that in humans and lacks *γ*2 isoform [2]. This may contribute to the differences observed in the lipid and glucose metabolism of chickens compared to mammals. In order to understand the dynamics of fat deposition in chicken, it is essential and valuable to have an understanding of lipid metabolism, morphology changes, and transcription of regulatory genes that are involved in chicken adipose tissues.

### 4.1. Fatty Acid Composition of the Abdominal Fat

In the present study, the content of* cis*-9,* trans*-11 CLA ranged from 1.69- to 2.3-fold greater (*P* < 0.05) than that of* trans*-10,* cis*-12 CLA in abdominal fat of LCLA and HCLA groups.* cis*-9,* trans*-11 CLA or* trans*-10,* cis*-12 CLA contents increased linearly with increasing the level of CLA in the feed. These indicated that the CLA content in the feed influences the fatty acid composition of abdominal fat, which was in line with Suksombat et al. [39] and Szymczyk et al. [40]. This could be due to the fact that* cis*-9,* trans*-11 and* trans*-10,* cis*-12 CLA isomers are metabolized at different rate in the peroxisomes. This may contribute to the lower accumulation of* trans*-10,* cis*-12 isomers into tissues [41]. The overall CLA content in chicken abdominal fat increased from 0 to 1.30 and to 4.07 in CON, LCLA, and HCLA, respectively.

The current study clearly showed that CLA-fed chickens experienced an increase in SFA and a decrease in MUFA contents within their abdominal fat. This is consistent with findings by Aydin [42] among pigeons fed a diet containing CLA and in rats [43, 44]. The increase of SFA at the expense of MUFA most likely resulted from the inhibition of the Δ-9 desaturase enzyme system that is responsible for SFA desaturation, converting SFA into MUFA [45]. Our data also showed that most of PUFA, particularly the long-chain n-3 PUFA, were affected by dietary CLA supplementation. The increase of n-3 PUFA might be a result of higher Δ5 and Δ6 fatty acyl desaturase activity [46]. The linoleic acid (C18:2 n-6) and arachidonic acid (C20:4 n-6) levels were not significantly different in abdominal fat tissues of CLA-fed chickens. This is most likely due to CLA having similar alteration as linoleic acid (LA) but with subtle isomer variances, which may decrease the deposition of LA in adipose tissue rich in neutral lipid where CLA is preferentially accumulated [41]. Thus, the conversion of LA into arachidonic acid (AA) would decrease eventually. Furthermore, Du et al. [47] observed a decrease in C18:2 n-6, C18:3 n-3, and C20:4 n-6 in laying hens consuming 2.5% mixed CLA isomers compared to control. It has been suggested that reduction in adipogenic fatty acids such as LA and AA may reduce triglycerides content (which was not measured in this study) that is important for prostaglandin synthesis, which may regulate adipogenesis [48].

### 4.2. Transcriptional Expression of PPAR Genes

Based on the results in Table 1, the CLA clearly affected the fatty acid composition of abdominal fat. This is particularly true for the adipocyte, which showed decreased degree of fatty acid unsaturation. It is probable that this shift in fatty acid profile may be related to modification of membrane adipose tissue by CLA through alteration of adipocyte gene and decrease in the concentration and activity of the Δ-9 desaturase enzyme [49].

Among the genes that appeared to be relevant to the effects of CLA is the PPAR [23]. Many reports indicated that PPAR*γ* is a component of adipocyte transcription and differentiation. PPAR*γ* regulates lipid homeostasis, which in turn controls the transcription genes that were involved in cellular metabolism and differentiation [22, 50]. In this study, CLA-fed chickens exhibited decreased mRNA levels of PPAR*γ* in abdominal fat (*P* < 0.05). Similar observations were noted by Granlund et al. [51], where they reported that the downregulation of the PPAR*γ*, C/EBP, and aP2 transcription in* 3T3-L1* preadipocyte by CLA mixed isomers is responsible for the attenuation preadipocyte differentiation. Both CLA isomers have low affinity for PPAR*γ* compared to PPAR*α* [40]. This may suggest that CLA might exhibit little or no effect on adipose tissue, a process that is clearly mediated by PPAR*γ* [52]. Thus, CLA could mediate by reducing PPAR*γ* transcription in preadipocytes and adipocytes [21]. Alternatively, studies have shown that CLA could actually operate as a PPAR*γ* antagonist [53]. This explains the mechanism of CLA being able to alter adipose tissue without being a potent PPAR*γ* agonist.

Most of the PPAR*γ* target genes in adipose tissue are directly implicated in lipogenic pathways, for example, aP2, which involved the uptake and transport of fatty acids in adipose tissue. The aP2 gene contains a PPAR-response element (PPRE) [54] and CLA is a ligand for PPAR*γ*. In the present study, CLA may have elevated aP2 transcription, which is parallel to PPAR*γ* transcription in adipose tissue of broiler chickens. These observations indicated that CLA could also function through PPAR*γ*. In fact the aP2 gene is regulated by PPAR*γ* [17] which induces adipocyte differentiation as PPAR*γ* is regarded as “master regulator” of adipocyte differentiation.

Our data indicate that PPAR*α* transcriptional level was not significantly affected by dietary CLA intake (*P* > 0.05). PPAR*α* is poor inducer of adipogenesis [55]. Our findings echoed that of Peters et al. [56], suggesting that PPAR*α* may not be a pivotal transcription factor for adipose tissue.

### 4.3. Abdominal Fat Cellularity

The results of abdominal fat cellularity study demonstrated the distinct effects of dietary CLA supplements on the number, diameter, and area of adipocytes. MUFA is associated with adipocytes area, which underlines the theory of a differential metabolic and desaturase activity. Considering that Δ-9 is the key enzyme converting SFA into MUFA and bearing in mind the close association between most MUFA and adipocytes area, it would be plausible to speculate that higher adipocytes area found in the subcutaneous fat is responsible for a higher desaturation activity [57]. In our study, CLA supplemented diet containing low MUFA, which correlates with reduced number of fat cells. Reduction of mean adipocyte size is an effective approach to reduce body fat. Smaller mean abdominal adipocyte volume yields a smaller amount of fat. This decrease in mature abdominal adipocytes may be responsible for the reduced adipose tissue size. In fact, because of adipocyte precursor cells' (i.e., preadipocytes) presence throughout the life, inhibition of the fattening process may be mediated not only by reducing body fat accumulation in differentiated adipocytes, but also by inhibiting the differentiation of preadipocytes into adipocytes. This hypothesis is supported by several papers, carried out in* 3T3-L1* cell culture, which has shown that CLA inhibit adipocyte differentiation [58, 59].

Our results showed that 5% CLA included in the diet decreased the abdominal fat cell numbers. The reduction of abdominal adipocyte number in chickens may be the result of the reduction of abdominal adipose precursor cells caused by CLA isomer. Sisk et al. [27] demonstrated that CLA reduced adipocyte volume in* Sprague-Dawley* rats sufficiently to account for the reduction in adipose tissue mass. Our data suggest that CLA could depress body fat accumulation by reducing preadipocyte number. Preadipocytes might be a target for inhibition of differentiation, in which CLA may cause a decrease in the number of cells potentially able to become mature adipocytes, thus indirectly diminishing bodily fat mass. Brandebourg and Hu [60] have recently observed that the CLA isomer inhibits porcine preadipocyte differentiation by a mechanism that involves the downregulation of PPAR*γ* mRNA. The CLA isomers also decrease preadipocyte differentiation by downregulating the peroxisome PPAR*γ* transcription in humans [50]. Taken together, our data suggested that CLA downregulated PPAR*γ* and aP2, which subsequently resulted in a decrease in adipocyte size, number, and area of abdominal fat cells.

## 5. Conclusions

Taken together, it could be concluded that the relative transcriptional pattern of adipogenic genes and cellularity characteristics of adipose tissue were influenced by CLA. Results also showed that* cis*-9,* trans*-11 CLA, rather than* trans*-10,* cis*-12 CLA, is the dominant isomer in the abdominal fat pads of LCLA and HCLA groups. CLA treated groups had lower transcriptional level of PPAR*γ* and aP2. This was associated with lesser mean abdominal adipocyte volume and a smaller amount of fat as a result of reduced capacities to store fats. It is also apparent that the transcription of key adipogenic genes and adipose cellularity played a role in enabling the dietary CLA to influence fatty acid composition of adipose tissues in broiler chickens.

## Figures and Tables

**Table 1:** Ingredient and chemical composition of diet.

Ingredient (% DM)	Starter (1–21 days)			Finisher (22–42 days)		
	CON	LCLA	HCLA	CON	LCLA	HCLA
Corn	51.17	51.17	51.17	58.9	58.9	58.9
Soybean	40.56	40.56	40.56	32.22	32.22	32.22
Palm oil	5	2.5	—	5	2.5	—
Common salt	0.4	0.4	0.4	0.4	0.4	0.4
^ 1^Vitamin premix	0.05	0.05	0.05	0.05	0.05	0.05
^ 1^Mineral premix	0.05	0.05	0.05	0.05	0.05	0.05
DL-methionine	0.26	0.26	0.26	0.3	0.3	0.3
Lysine	—	—	—	0.5	0.5	0.5
^ 2^Chemical composition						
Crude protein (% DM)	22.00	22.00	22.00	20.5	20.5	20.5
Metabolizable energy (ME) (Kcal/kg)	3080	3080	3080	3150	3150	3150
Phosphorus (% DM)	0.45	0.45	0.45	0.42	0.42	0.42
Calcium (% DM)	1.00	1.00	1.00	0.9	0.9	0.9
Methionine (% DM)	0.55	0.55	0.55	0.5	0.5	0.5
Lysine (% DM)	1.20	1.20	1.20	1	1	1
Na (% DM)	0.20	0.20	0.20	0.15	0.15	0.15

CON: control; LCLA: low conjugated linoleic acid; HCLA: high conjugated linoleic acid.

^1^Premixes contributed the following nutrients per kilogram of complete feed: vitamin A, 2300 IU; vitamin D_3_, 400 IU; vitamin E, 1.8 mg; vitamin B_12_, 3.5 mg; riboflavin, 1.4 mg; pantothenic acid, 2 mg; nicotinic acid, 7 mg; pyridoxine, 0.25 mg; folic acid, 0.15 mg; menadione, 0.3 mg; thiamin, 0.15 mg; manganese oxide, 35 mg; ferrous sulfate, 35 mg; zinc oxide, 30 mg; copper sulfate, 60 mg; cobalt carbonate, 5 mg; potassium iodine, 0.6 mg; selenium vanadate, 0.09 mg. CLA used in this study was Lutrell pure, BASF, Germany, which contained 60% of both CLA isomers. Dietary inclusion of CLA 5% and 2.5% will be equal to 3.0% and 1.5% of both CLA isomers respectively.

^2^Calculated values.

**Table 2:** Fatty acid composition (g/kg feed) of experimental diets.

Fatty acids	Starter (1–21 days)			Finisher (22–42 days)		
	CON	LCLA	HCLA	CON	LCLA	HCLA
C12:0	0.73	0.48	0.23	0.76	0.51	0.26
C14:0	3.19	1.69	0.19	3.23	1.72	0.22
C16:0	23.00	15.72	8.43	23.34	16.06	8.78
C16:1	0.53	0.28	0.03	0.53	0.28	0.03
C18:0	2.71	14.52	26.32	2.81	14.62	26.42
C18:1n-9	29.74	23.53	17.30	31.33	25.12	18.90
C18:2n-6	12.00	10.05	8.03	12.18	10.20	8.20
C18:3n-3	0.79	0.54	0.29	0.82	0.57	0.32
*cis*-9, *trans*-11 CLA	0.00	2.97	5.97	0.00	3.00	5.95
*trans*-10, *cis*-12 CLA	0.00	2.92	5.90	0.00	2.93	5.91
^ a^Total SFA	29.63	32.41	35.18	30.13	32.91	35.69
^ b^Total MUFA	30.27	23.81	17.34	31.86	25.40	18.94
^ c^Total n-3 PUFA	0.79	0.54	0.29	0.82	0.57	0.32
^ d^Total n-6 PUFA	12.00	10.05	8.03	12.18	10.20	8.20
Total PUFA	12.79	10.59	8.33	13.00	10.77	8.52
^ e^n-6 : n-3FAR	15.16	18.54	27.53	14.77	17.75	25.27
PUFA : SFA	0.43	0.33	0.24	0.43	0.33	0.24
^ f^Total CLA	0.00	5.89	11.86	0.00	5.93	11.86

CON: control; LCLA: low conjugated linoleic acid; HCLA: high conjugated linoleic acid.

^a^Total SFA = sum of C12:0 + C14:0 + C16:0 + C18:0.

^b^Total MUFA = sum of C16:1 + C18:1n-9.

^c^Total n-3 PUFA = sum of C18:3n-3.

^d^Total n-6 PUFA = sum of C18:2n-6.

^e^n-6 : n-3 fatty acid ratio (FAR) = sum of C18:2n-6 ÷ sum of C18:3n-3.

^f^Total CLA = sum of *cis*-9, *trans*-11 CLA + *trans*-10, *cis*-12 CLA.

**Table 3:** Sequences of forward and reverse primers for real-time PCR.

Genes	Sense primer (5′-3′)	Size	Annealing temperature	Reference
PPAR*α*	F-AGGCCAAGTTGAAAGCAGA R-GTCTTCTCTGCCATGCACAA	217	60	K$\ddot{\text{o}}$nig et al. [61]
PPAR*γ*	F-GACCTTAATTGTCGCATCCAT R-CGGGAAGGACTTTATGTATGA	237	61	Zhang et al. [62]
aP2	F-GAGTTTGATGAGACCACAGCAGA R-ATAACAGTCTCTTTGCCATCCCA	107	63	Sato et al. [63]
GAPDH	F-TGAAAGTCGGAGTCAACGGATT R-CCACTTGGACTTTGCCAGAGA	81	60	Ojano-Dirain et al. [64]
*β*-Actin	F-ATGAAGCCCAGAGCAAAAGA R- GGGGTGTTGAAGGTCTCAAA	223	62	K$\ddot{\text{o}}$nig et al. [61]

F: forward.

R: reverse.

**Table 4:** Fatty acid profile of the abdominal fat of broiler chicken (percentage of total identified fatty acids) across treatment groups.

Fatty acids	CON	LCLA	HCLA	SEM	*P* value
C12:0	0.10	0.18	0.23	0.039	0.379
C14:0	0.97^b^	0.91^b^	1.11^a^	0.028	0.004
C16:0	29.54^b^	28.88^b^	32.39^a^	0.500	0.002
C16:1	4.22	4.03	3.51	0.160	0.143
C18:0	7.80^b^	7.32^b^	14.16^a^	0.755	<0.0001
C18:1n-9	40.67^a^	40.30^a^	27.96^b^	1.370	<0.0001
C18:2n-6	16.03	15.81	15.33	0.199	0.305
C18:3n-3	0.44^b^	0.47^b^	0.89^a^	0.057	<0.0001
*cis*-9, *tran*-11 CLA	0.00^c^	0.92^b^	2.56^a^	0.214	<0.0001
*trans*-10, *cis*-12 CLA	0.00^c^	0.39^b^	1.51^a^	0.131	<0.0001
C20:4n-6	0.21	0.78	0.35	1.404	0.197
^ a^Total SFA	38.42^b^	37.30^b^	47.88^a^	1.230	<0.0001
^ b^Total MUFA	44.90^a^	44.34^a^	31.47^b^	1.472	<0.0001
^ c^Total n-3 PUFA	0.44^b^	0.47^b^	0.89^a^	0.057	<0.0001
^ d^Total n-6 PUFA	16.25	16.59	15.68	0.217	0.192
Total PUFA	16.69	17.06	16.57	0.223	0.632
^ e^n-6 : n-3 ratio	36.93^a^	35.29^a^	17.61^b^	7.392	0.033
PUFA : SFA ratio	0.45^a^	0.46^a^	0.35^b^	0.001	0.0007
^ f^Total CLA	0.00^c^	1.30^b^	4.07^a^	0.344	<0.001

CON: control; LCLA: low conjugated linoleic acid; HCLA: high conjugated linoleic acid.

The data are expressed as the percentage of total identified fatty acids.

^a^Total SFA = sum of C12:0 + C14:0 + C16:0 + C18:0.

^b^Total MUFA = sum of C16:1 + C18:1n-9.

^c^Total n-3 PUFA = C18:3n-3.

^d^Total n-6 PUFA = sum of C18:2n-6 + C20:4n-6.

^e^n-6 : n-3 FAR = sum of (C18:2n-6 + C20:4n-6) ÷ (C18:3n-3).

^f^Total CLA = sum of *cis*-9, *trans*-11 CLA + *trans*-10, *cis*-12 CLA.

Data presented as mean with pooled SEM (*n* = 10). ^a,b^Mean values within a row with no common superscript differ significantly (*P* < 0.05).
